# Shifting educational gradients in body mass index trajectories of Indonesians: an age period cohort analysis

**DOI:** 10.1186/s12889-022-13379-3

**Published:** 2022-05-18

**Authors:** Lilipramawanty Kewok Liwin

**Affiliations:** School of Demography, The Australian National University, Canberra, Australian Capital Territory Australia

**Keywords:** Educational gradients, BMI trajectories, Age period cohort effects, Multi-level modeling, And longitudinal analysis

## Abstract

**Background:**

Globally, the number of obese adults has increased rapidly in many developing countries. The links between increased educational attainment and lower risks of overweight/obesity have been studied in a number of high-income contexts. However, educational attainment can have a different association with obesity at different levels of economic development and different stages of the nutritional transition, and these associations may vary by period and cohort. This study aims to provide evidence on the shifting of educational gradients in overweight/obesity in Indonesia, a low middle income country.

**Methods:**

Using five waves of Indonesian Family Life Survey (IFLS), this study examines the Body Mass Index (BMI) trajectories of 14,810 individuals from 1993 to 2014. This study analyses how educational gradients in BMI have shifted over time and across cohorts using a hierarchical age-period-cohort (HAPC) model to account for the effects of age and the changes in historical periods (social and environmental contexts).

**Results:**

In older generations, higher educational attainment is associated with higher BMI, but the gap between educational groups shrinks in more recently-born cohorts. The BMI of lower educational groups is catching up with that of the tertiary educated, leading to an increased risk of overweight/obesity among low educated individuals. Having tertiary education lowers the risk of weight gain (-0.04 point) among recently-born cohort of women, but it still increases the risk (+ 0.04 point) for men.

**Conclusion:**

Changes in access to education and the ongoing nutritional transition in Indonesia are leading to a shifting of educational gradients in overweight/obesity over time. The rising trends in BMI among low-educated and younger individuals are of substantial concern for Indonesian public health due to their implications for the risk of communicable and non-communicable diseases in the future.

**Supplementary Information:**

The online version contains supplementary material available at 10.1186/s12889-022-13379-3.

## Introduction

### Global obesity epidemic

Adult obesity is a global public health challenge and has indirect implications for countries’ economic development. Literature has documented the association between overweight/obesity with all-cause mortality [1]. Overweight/obesity has been established as a major risk factor for morbidity from Non-Communicable Diseases (NCDs) including high blood pressure, cardiovascular diseases, and diabetes [2–4]. The high cost of obesity-related diseases results in a substantial burden to health systems and society [5]. Further, overweight/obesity has indirect costs to economic growth due to the high rates of sick leave, disability, and premature mortality among obese workers leading to productivity loss [6].

Health institutions and scholars have been sounding the alarm on the global rise of overweight and obesity. A multi-country study has shown the continued rise in adults’ Body Mass Index (BMI) in most countries in the world [7]. Obesity is projected to affect one in five adults globally by 2025 with predicted prevalence 18% in men and above 21% in women [8]. Obesity has been commonly understood as a public health challenge in high-income countries, but the rate of increase in overweight and obesity is much faster in low- and middle-income countries [7, 8]. The top 10 countries with the most rapid increase in obesity prevalence during 1995–2016 are located in the Asia–Pacific Region including Laos, Vietnam, Indonesia, and Timor-Leste [8]. Underscoring the serious public health implications of the obesity epidemic, the WHO calls for countries to prevent the rise in obesity prevalence by 2025. However, the Global Obesity Federation has reported that most countries are falling short in halting the rise in obesity [8].

Although the prevalence of overweight and obesity are rising globally, the epidemic is not evenly distributed across population groups. Previous studies shown notable gender, socioeconomic, ethnic, and spatial/geographical disparities in the prevalence of overweight/obesity within and across countries [9–12]. These social inequalities reflect differences in the quality of living conditions, which in turn affects the opportunity to pursue a healthy life and maintain optimum weight. Further, it has been suggested that environmental exposures during specific periods may contribute to social gradients in health outcomes in certain populations [13, 14]. Reversing this rise of global obesity requires intervention beyond individual responsibility to maintain optimum weight. Hence, there is a need to understand social determinants of health contributing to the disparity within and between countries, in particular in low-middle income countries.

### Heterogeneous educational gradient of obesity

Education is a fundamental social determinant of health outcomes, and is associated with better health and lower risk of poor health behaviors [15, 16]. The close link between education and obesity is also notable [17]. However, the relationship between education and obesity manifests in different ways in different population contexts. A systematic review found differences in both the magnitude and direction of the association between education attainment and obesity, and found that these effects are modified by gender and level of economic development [17]. Studies in most high-income countries found that more educated individuals have a lower risk of obesity [17, 18]. The opposite association was often observed in lower-income countries—with more educated individuals at a higher risk of obesity [19]. Baker et al. [20] have argued that these different manifestations of the relationship between education and obesity suggest that the effect of education on obesity is contextual, varies over time, and is closely related to the nutritional transition across populations.

Examination of the relationship between education and health has challenges because access to, and the quality and value of, education changes over time in ways that may affect the association. Globally, there has been a significant decline in educational inequality [21], meaning that younger cohorts tend to have better access to education as compared to older cohorts. It has been suggested these changes in education may have substantial effects on the education-obesity relationship over time [22]. Thus, there are likely to be complex age, period, and cohort effects on the relationship between education and obesity that need to be investigated [9, 17].

Despite many studies that have investigated the association between education and health, there have been few studies investigating whether the relationship between education and health varies over life course and across birth cohorts [22]. Ignoring age and cohort effects on the relationship between education and health can lead to incorrect conclusions or biased estimation of the true underlying relationship using both cross-sectional and longitudinal data [22]. Therefore, careful examination is needed in considering the effect of education on changes in BMI across cohorts. Further, few studies have been considered how changing access to education across cohorts may affect educational gradients to BMI.

In addition, most published literature on education gradients and obesity using longitudinal data has come from developed countries, and research specifically investigating the effect of education on BMI remains scarce in developing countries [17, 23, 24]. The available evidence in low- and middle-income counties is almost entirely based on cross-sectional data. Such cross-sectional data can only measure social differences in obesity at a single point of time based on the distribution of observable characteristics [25]. However, BMI is not a fixed characteristic, and instead varies within individuals across their life course. As such, cross-sectional analyses have only limited ability to examine how characteristics such as education attainment are linked to an individual’s changing risk of obesity over the life-course.

The present study aims to fill this important gap in the literature on individual BMI trajectories over the life-course by investigating how the association between education and BMI changes across cohorts in a low-middle income setting. Further, this research explores potential factors that may modify the association between education and BMI by applying an age-period-cohort model.

### Environmental change and the overweight/obesity epidemic

It has been suggested that social gradients of weight gain are also associated with social and environmental contexts, which vary across time [25]. The obesity epidemic is fueled by environmental changes known as obesogenic environment that affects the whole population [26–28], for instance the changes in economic growth, policies aiming to improve food supplies and accessibility, automatisation leading to a more sedentary lifestyle, social norms, urban designs lacking in recreation space, and the proliferation of fast food availability. Changes in this obesogenic environment expose all social groups to the risk of obesity, but in ways that depend on individual genetic, socioeconomic, and sociocultural factors [26, 27]. Changes in the environment also affect men and women differently. Sociocultural contexts drive gender differences in dietary intake, physical activities, and gendered cultural values favoring large body shapes [29]. During the nutritional transition, the increase in food availability and energy-dense foods worsen sociocultural effects on gender disparities in obesity [29].

Sturm and An [26] have suggested that the study of change can provide insight on the effect of the environment on the obesity epidemic, and how the effect of obesogenic changes on the risk of obesity differs across social groups. Longitudinal data allows researchers to investigate how the rate of change of an outcome may vary across population groups [30]. Hence, longitudinal data tracking individuals’ body mass index (BMI) over time permits investigation into the impact of historical period effects (obesogenic changes) on individual BMI trajectory embedded in birth cohorts and period of observation.

However, there are methodological challenges to testing cohort effects in the study of social change due to potential collinearity issues between period, cohort and age as Age = period-cohort [31]. Siegel and Olshansky [31] have argued that Age-Period-Cohort (APC) should be examined jointly to prevent errors of interpretation. The hierarchical age-period-cohort (HAPC) model has been proposed to jointly explore APC effects [32]. This present study applies HAPC to test cohort effects on BMI trajectory.

### Indonesian context

Indonesia is the world’s fourth most populous nation with about 270 million people, and is classified as a newly industrialized country. Indonesia’s economic growth from 1900 to 2010 was characterized by instability as a result of economic and political changes within the country, including the Great Depression in the 1930s, colonization prior to independence in 1945, political instability post-independence, and the 1997–98 Asian financial crisis [33]. This economic history may leave its mark on the quality of life of individuals in the country over time. Based on data collected from Indonesia military recruits who were born from 1890 to 1990, there is a downward trend in Indonesian height among those who were born in 1930 to early 1940s, and an upward trend for those who were born after 1950 [33]. This shows evidence of an important historical effect related to birth cohort, which may have its implications for individual’s later development. Hence, examining the effect of birth cohort and period when individuals were observed are essential to understand trend of obesity in Indonesia.

Consistent with the global rise in overweight/obesity, Indonesia has experienced a rise in the proportion of the overweight or obese population in all ages over the past two decades. The Indonesia National Basic Health Research Survey (Riskesdas) has observed a rapid increase of overweight and obesity among adults age over 18 years old from 21.7% (combined overweight and obese) in 2010 to 35.3% in 2018 [34, 35].

Prior evidence from Indonesia found heterogeneity in the prevalence of overweight/obesity by age, gender, education status, income level, marital status, and urban/rural residential areas [34, 36–38]. Recent studies on BMI in Indonesia have shown narrowing inequalities in the overweight/obesity distribution and a fairly even dispersal across socioeconomic groups [37, 39, 40]. Overweight and obesity has been rising rapidly among the poor, people with low education, and in rural populations [40].

Previous literature examining the trend of overweight/obesity in Indonesia have relied almost entirely on cross-sectional analysis. The small number of studies using longitudinal data to explored changes in mean BMI across population groups [38, 40] did not consider variation on individual BMI growth patterns over the lifespan. To our knowledge, no previous study has used repeated measures of BMI to model BMI trajectories, and explore how BMI changes with age from early to late adulthood in Indonesia. By taking into account intra- and inter-individual differences in BMI changes over life-course, this study aims to identify how the trajectory of BMI differs by sex and cohort in this rapidly changing context.

In addition, although previous studies have investigated the differences in BMI by level of education, none have specifically investigated educational gradients of BMI trajectories, nor have examined how changes in access to education across birth cohorts shape BMI trajectories [36–38]. In fact, inequality in access to education in Indonesia has decreased over time [41], meaning younger generations have experienced greater access to higher education than previous generations. Despite these improvements, Census 2010 shows that gender and household economic differences in access to education persist among cohorts of over 30 s [42]. These changes in access to education may affect the association between education and obesity. Further, the education-obesity relationship may manifest in different ways for men and women due to the gender gap in access to education and sociocultural context of the country.

### Research questions

Using longitudinal analysis, this study attempts to understand the rising burden of obesity in low-middle income setting by examining individuals’ BMI trajectories and the social gradient of weight gain in Indonesia. This research will test Baker et al.’s [20] hypothesis on “an expected shifting in the direction of education to new risk behaviours across educational spectrum” as result of “macro-level environmental influences”. This research is guided by three key research questions:

1. Are there distinct sex differences in individuals’ BMI trajectories over age?
2. What are the age, period, and cohort patterns of BMI trajectories in Indonesia?
3. How does the education-BMI relationship change over age and across cohorts?

## Methodology

### Data source

Using longitudinal data from the Indonesian Family Life Survey (IFLS), this study examines how individual BMI trajectories have changed over two decades (from 1993 to 2014). There are five waves of IFLS until 2014, and data is available for public use at https://www.rand.org/labor/FLS/IFLS.html. During two decades of observations, there were new panel respondents added in each wave. This present study includes both original and new panel respondents in the analysis to increase sample power. Analysis in this study is conducted without longitudinal sample weighting because IFLS datasets only generated individual longitudinal weighting for original respondents who had been followed since the first wave.

This study focuses on examining changes in BMI among adults born prior to 1974, who were aged 20 or over at the first wave of IFLS data collection in 1993. IFLS followed and collected information of households and individual respondents over time, including anthropometric measures (height and weight) from all individual respondents that are used to measure BMI. As a household survey, IFLS followed individual BMI from a wide range of cohorts over 20 years that allows examination of individual weight change as function of age, as well as the cohort shift in mean BMI over time.

Pregnant women are excluded from observation to reduce bias analyzing individual BMI trajectory. This present study also excludes observations with extreme outliers of height (< 100 cm or > 200 cm) and weight (< 25 kg or > 200 kg). Overall, there are less than 0.6% of observations excluded due to extreme height and weight (see Supplemental Table 1). To examine the change in BMI over time, this study only includes panel respondents with a minimum of three measures of BMI to permit more flexible BMI trajectory modelling. Supplemental Table 2 provides participation rates of each birth cohort under study.

### Outcome: Body Mass Index (BMI)

The outcome interest of this study is BMI, which is a continuous time-varying variable. BMI is a general indicator to measure and classify individual adults considered as underweight, normal, overweight or obese. BMI is calculated from individual weight in kilograms divided by the square of the person height in meters (kg/m^2^) [43]. The risk of chronic diseases among Asian populations is increased at lower BMI cut points as compared to international BMI cut points (≥ 25–29.9 kg/m^2^ for overweight and ≥ 30 kg/m^2^ for obese) [44]. Thus, the cut points for Asia–Pacific population are 23–27.4 kg/m^2^ for overweight and ≥ 27.5 kg/m^2^ for obese [43, 44].

### Covariates

To explain variation in BMI trajectories across population groups, this study will examine whether BMI trajectory differs by age, sex, cohort, period and educational attainment over time.

#### Educational attainment

This study investigates the effect of the highest education attained after the age of 20 in shaping BMI changes across the population. Data exploration finds that only 1.2% of panel respondents experienced changes on educational status over two decades of IFLS observation. Hence, education is treated as a time invariant variable with the assumption the level of educational attainment of panel respondents is unchanged after the age of 20. Level of education attainment is treated as a categorical variable with 1 = no education, 2 = primary school, 3 = secondary school (junior and senior schools), and 4 = tertiary.

#### Control variables: age, sex, period and birth cohorts

In this analysis, the individual’s age in each wave represents time and is treated as a continuous variable. Age is centered at 20 in the models to facilitate meaningful interpretation of the intercept. By observing how BMI changes as a function of age, this study can reveal the true effect of age on BMI trajectories. Sex is measured as a dummy variable (0 = men and 1 = women). Based on previous evidence, we hypothesize that BMI trajectories may differ substantially by sex. To account for the effect of historical period when the surveys were conducted, a five-category period variable (1 = 1993, 2 = 1997, 3 = 2000, 4 = 2007, and 5 = 2014) is used. A continuous cohort variable is created to estimate the effect of birth cohort. The cohort variable is also centered at youngest cohort of panel respondents (respondents born in 1973). All available cohorts are used in this analysis, so there are differences in number of cohort groups between women (70 groups) and men (72 groups).

### Statistical analysis

This study fits an individual growth model, and examines BMI trajectories using a mixed-effects approach to Growth Curve Modelling (GCM) that permits estimation of inter-individual variability by taking account of intra-individual trajectories of change over time [45]. This model can fit unbalanced data, meaning that individual BMI trajectories can contain a unique number of BMI measures which were collected at unique times [30]. The mixed-effect approach also can fit complex models containing two or three-level random-effects without difficulty [46].

To describe the growth curve model, the three level model is defined as follows:

$${y}_{ijk}={\beta }_{0}+{\beta }_{1}({Age}_{ijk})+{\beta }_{n}{X}_{ijk}+{\upsilon }_{0j}+{\upsilon }_{1j}({Age}_{ijk})+{\gamma }_{k}+{\varepsilon }_{ijk}$$

The outcome ${y}_{ijk}$ represents BMI measures at time i, of individual panel respondent j, of k birth cohort. The ${\beta }_{0}+{\beta }_{1}({Age}_{ijk})+{\beta }_{n}{X}_{ijk}$ represents the fixed portion of the model with ${\beta }_{0}$ for population average of BMI intercept, ${\beta }_{1}({Age}_{ijk})$ for estimated effect of age on average in BMI slope, and ${\beta }_{n}{X}_{ijk}$ for estimated effect of other covariates (including: age^2^, sex, period, birth cohort, education level and their interactions). The ${\upsilon }_{0j}+{\upsilon }_{1j}({Age}_{ijk})+{\gamma }_{k}$ illustrates the random effect of the model with ${\upsilon }_{0j}$ as the random intercept of BMI between individuals, ${\upsilon }_{1j}({Age}_{ijk})$ as the random slope of BMI changes as a function of age between individuals, and ${\gamma }_{k}$ as the random intercept of BMI between birth cohorts. Then ${\varepsilon }_{ijk}$ represents error term.

This study comprises two steps of analysis. First, we model trajectories of BMI and examine whether there is a significant variation within and between persons in BMI changes over the life-course. We further investigate whether the association between age and BMI follows a quadratic or curvilinear trend over age. The analysis will also test whether BMI trajectory differs by sex over the life-course. The mixed model consists of two-level random effects models that count for intra- and inter-individual differences in BMI trajectory. Sensitivity analysis including all observations with a minimum of one BMI measure shows consistent direction on the association between covariates and BMI trajectory (see Supplemental Table 3).

In the second step of analysis, we develop separate models for men and women to investigate the effect of age, period, and cohort on changes in population BMI. These analyses additionally investigate how educational gradients in BMI trajectory have changed over time after controlling for period and cohort effects. Due to significant differences in access to education and in BMI trajectories between men and women, these analyses are conducted separately by sex.

This paper adopts the hierarchical age-period-cohort model (HAPC) by Bell and Jones [32] to model age-period-cohort effects on BMI trajectories. Using HAPC analysis, the trend in BMI of the Indonesian population is evaluated according to three different dimensions of time: age (life course), period (historical time when the survey occurred), and birth cohort (historical effect of the year in which individuals were born). The HAPC model has the flexibility to estimate linear and other polynomial effects of APC in the fixed part of the model, while simultaneously take account for discrete effect of cohort or period in the random part, allowing for meaningful analysis on how APC effects operate [47]. However, the model does not necessarily solve the identification problem and may apportion APC effects based on the data structure rather than actual process of APC to outcome of interest [47]. Hence, a combination of justified theoretical foundation, evidence from previous literature and the data gathering pattern may lead to plausible estimation on the effect of age, period, and cohort.

The data structure of the ILFS comprises a wide range of birth cohorts who were observed through 5 waves from 1993 to 2014. Based on the data structure, the effects of APC are investigated simultaneously by the inclusion of age, period, and cohort as covariates at fixed model. The mixed model with APC in this analysis is a three-level random-effects model taking account intra-and inter-individual differences in BMI changes at second level, as well as intra-and inter-cohort differences at the third level. The model also includes linear age as an individual random effect to allow the effect of age on BMI trajectory to vary across individuals.

The main effect of education is examined by including education covariates in the fixed model. Further, interactions between education and age and/or cohort are included to test differences in the effect of education over the life-course and across cohort after controlling for the historical period. Statistical analysis was carried out using Stata, version 14.2. As panel respondents included in the analysis have 3 to 5 measurements of their BMI, there are missing values of BMI for panel respondents who only contribute 3 or 4 measurements. Hence, to handle the missing BMI values, the models estimation apply a maximum likelihood method. A likelihood-ratio test in Stata 14.2 is performed to evaluate model fit.

## Findings

### Descriptive analysis

In total there are 14,810 panel respondents with three or more BMI measures included in this analysis. The distribution of panel respondents in each wave is illustrated by Table 1. Table 1 shows the unweighted distribution of observations in this study for each wave based on respondent characteristics (sex, age group, education attainment and BMI category). The table shows a gradual increase in the proportion of overweight/obese over time from 1993 to 2014. The mean BMI also increased from 21.4 kg/m^2^ in 1993 to 23.85 kg/m^2^ in 2014. Overweight and obesity were more common in women than men. In 1993, 31.8% women and 20.5% men on this study were overweight/obese based on Asian BMI classification (see supplemental Table 4). The trend has increase over time with 61.1% women and 42.4% men classified as overweight/obese for the most recent wave in 2014.

**Table 1:** Descriptive proportion of respondents by characteristics from 5 waves of IFLS in 1993–2014 (*N* = 14,810)

Characteristics	Waves (*N* = 14,810)				
	I—1993	II—1997	III—2000	IV—2007	V—2014
*Sex*					
Female	56.27	56.26	53.75	54.5	54.92
Male	43.73	43.74	46.25	45.5	45.08
*Age group*					
20–29	18.52	16.2	9.04	0.05	0
30–39	30.28	30.64	30.64	21.28	1.04
40–49	20.89	22.31	25.48	32.31	36.40
50–59	17.61	16.41	16.41	22.33	31.86
≥ 60	12.7	14.44	18.43	24.02	30.71
*Cohort*					
1964 -1973	18.52	27.71	30.77	34.43	39.16
1954 -1963	30.28	28.43	27.77	29.42	31.52
1944–1953	20.89	18.21	17.45	17.36	17.17
1934–1943	17.61	14.93	14.02	12.36	9.22
≤ 1933	12.7	10.73	9.98	6.43	2.91
*Highest education attainment*					
Never/Not completed primary education	21.95	19.83	18.64	16.97	14.03
Primary	51.25	49.07	48.09	48.32	49.71
Secondary	23.29	26.70	28.46	29.84	31.12
Tertiary	3.51	4.40	4.81	4.88	5.14
*Asian BMI classification*					
Underweight	17.16	15.90	16.40	13.27	11.18
Normal	55.96	52.65	49.03	42.13	36.08
Overweight	20.96	23.57	25.40	29.74	33.45
Obese	5.91	7.88	9.17	14.86	19.29

Table 2 provides cross-tabulations of the distribution of panel respondents by educational attainment based on respondent’s sex and birth cohort groups. The distribution of educational attainment varies substantially by sex and over birth cohorts. Women have lower educational attainment compared to men, but the gap shrinks over successive generations as education became more accessible over time. Thus changing access to education by sex across successive cohorts needs to be accounted for when exploring the association between educational attainment and BMI trajectories.

**Table 2:** Distribution panel respondents based on education attainment by sex and cohort from 5 waves of IFLS in 1993–2014 (*N* = 14,810)

	**Never/Not completed primary**	**Primary**	**Secondary**	**Tertiary**	**Total**
Sex ***					
Female	24.5	47.45	24.52	3.52	100
Male	11.06	48.78	33.69	6.48	100
Cohort—10 years interval ***					
1964–1973	5.52	41.1	46.11	7.27	100
1954–1963	12.05	56.45	26.48	5.03	100
1944–1953	17.73	53.88	24.08	4.31	100
1934–1943	36.7	45.4	15.28	2.62	100
< = 1933	53.5	40.13	5.53	0.84	100

Pearson’s chi-squared test *** p-value < 0.001

### BMI trajectories of men and women

Table 3 shows a summary model investigating differences in the BMI trajectories over age, for men and women. Age has significant effects on BMI trajectories, and follows a quadratic function. In early adulthood, BMI increased by 0.16 points with each year of age, but the rate of change in BMI declined by -0.002 points with increasing age leading to a declining trajectory of BMI by late adulthood. This decline of BMI at late adulthood demonstrates the effect of biological aging processes including sarcopenia [48].

**Table 3:** Model BMI trajectories by sex over life course

	Model BMI trajectory by sex with 95% CI	
# of obs	**60,531**	
# of groups	**14,810**	
**Fixed effect**		
Mean BMI (intercept) at age 20	19.218***	[19.070–19.365]
Rate of change by one unit of age	0.162 ***	[0.153–0.172]
Changing in rate by one unit of age	-0.002***	[-0.002-(-0.002)]
Sex-(ref group Male)	0.121	[-.074–0.315]
Interaction sex-age	0.067***	[0.054–0.079]
Interaction sex—age square	-0.001***	[-0.001-(-0.0003)]
**Random effect**		
Level I—within person		
variance residual	2.750	[2.706–2.794]
Levell II—between person		
variance initial BMI (intercept)	9.275	[8.823–9.751]
variance rate of change (slope)	0.013	[0.013–0.014]
cov(intercept & slope)	-0.073	[-0.087-(-0.059)]
*p*-value random effect		
**Goodness of fit**		
Log likelihood	-143,188.790	
AIC	286,397.600	
BIC	286,487.700	

^*^** *p*-value significant < 0.001

BMI trajectories are significantly different between women and men over age. The findings in Table 3 suggest that there are sex differences in the rate of change by age and in age-related declines BMI in later life. At age 20, mean BMI is 0.12 points higher for women as compared to men (*p* < 0.001). Weight gain over age was more rapid for women, with BMI growing by 0.07 points per year more for women as compared to men. However, women also saw more rapid age-related declines at higher ages, (as shown by the significant interaction between the sex and age^2^ terms).

**Fig. 1:**
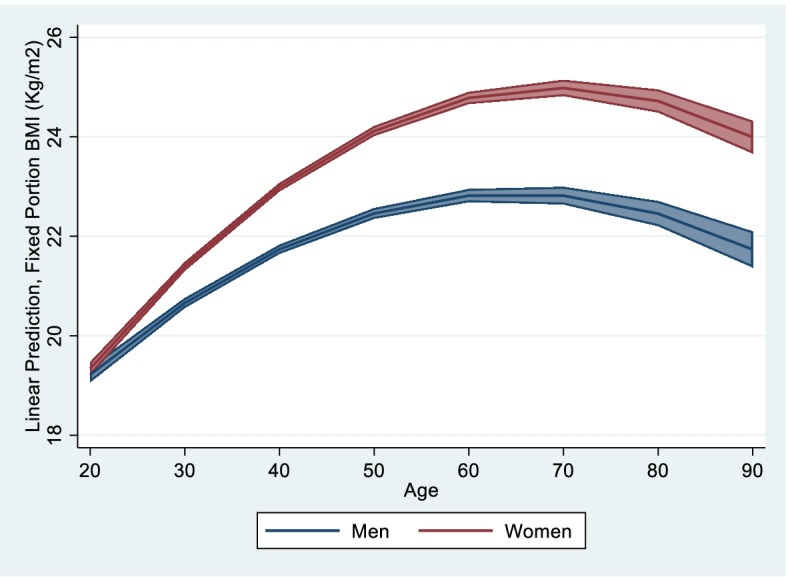
Predicted mean BMI trajectory for males and females over life-course with 95% CI

Predicted mean BMI over age adjusted for other covariates at means for women and men is illustrated on Fig. 1. The figure illustrates that women’s BMI increases faster than men at middle age, and women overall are more likely to be overweight after age 40 based on the Asia–Pacific BMI cut-off. Although men also saw increases in BMI over age, their average trajectory remains within the normal BMI spectrum.

Random effects from the above model show that individuals vary substantially in both their initial BMI (with variance = 9.28) and in the rate of increase in BMI over time (with variance = 0.01). The larger magnitude of variance on the BMI intercept as compared to the slope suggests that individuals’ initial BMI plays a major role in determining BMI trajectory over age between individuals.

Given the potential sex differences in the social, biological, and life-course drivers of BMI trajectories (including differences in access to education between men and women), this study estimates separate models for men and women. Separate models can reduce the complexity of analysis on testing the effect of education attainment over the life course and across cohorts.

### Age-period-cohort effects on BMI trajectories for women and men

Table 4 shows separate models for women’s and men’s BMI trajectories using Hierarchal Age-Period-Cohort growth curve modeling. The results suggest that age, period, and cohort independently affect the change of population mean BMI. The interaction between age, period and cohort is not significant, suggesting that there is no substantial correlation between APC effects.

**Table 4:** Summary table model for male and female

	**Female model with 95% CI**		**Male model with 95% CI**	
# of observations	**33,334**		**27,197**	
# of panel respondents	**8,003**		**6,816**	
# cohort group	**70**		**72**	
**Fixed effect**				
*Mean BMI (intercept) at age 20*	19.95***	[19.49–20.41]	19.40 ***	[18.94–19.85]
*Rate of change by one unit of age*	0.21***	[0.19–0.23]	0.17 ***	[0.15–0.18]
*Changing in rate by one unit of age indicating aging effect*	-0.003***	[-0.003-(-0.003)]	-0.002 ***	[-0.003-(-0.002)]
*Cohort (centering in cohort 1973)*	0.07***	[0.05–0.10]	0.06 ***	[0.04–0.08]
*Period of survey –ref 1993*				
1997	0.34***	[0.26–0.43]	0.03	[-0.04–0.11]
2000	0.38***	[0.26–0.50]	0.02	[-0.09–0.13]
2007	0.98***	[0.77–1.19]	0.51 ***	[0.32–0.71]
2014	1.49***	[1.19–1.78]	0.82 ***	[0.54–1.10]
*Education (ref. None education)*				
Primary	0.70***	[0.25–1.15]	0.30	[-0.18–0.78]
Secondary	0.21	[-0.27–0.70]	0.83***	[0.35–1.31]
Tertiary	-0.61	[-1.42–0.19]	1.91***	[1.28–2.53]
*Education#cohort*				
Primary	-0.01	[-0.03–0.01]	-0.005	[-0.02–0.01]
Secondary	-0.06***	[-0.08-(-0.03)]	-0.04***	[-0.06-(-0.02)]
Tertiary	-0.12***	[-0.18-(-0.06)]	-0.05***	[-0.08-(-0.02)]
*Education#Age*				
Primary	0.01	[-0.005–0.017]		
Secondary	0.04***	[0.02–0.05]		
Tertiary	0.06***	[0.04–0.09]		
**Random effect**				
Level I—within person variance	3.27	[3.21–3.35]	2.07	[2.02–2.12]
Level II—between person				
variance initial BMI (intercept)	10.99	[10.29–11.74]	6.59	[6.10–7.13]
variance rate of change (slope)	0.01	[0.01–0.01]	0.01	[0.01–0.01]
Cov (intercept & slope)	-0.12	[-0.14–0.10]	-0.07	[-0.09-(-0.06)]
Level III-between cohorts	0.16	[0.08–0.34]	4.09E-12	[6.6e-17–2.5e-07]
**Goodness of fit**				
Log likelihood	-80,852		-59,833.76	
AIC	161,748		119,705.5	
BIC	161,933.1		119,861.5	

*** *p*-value significant < 0.001

**Fig. 2:**
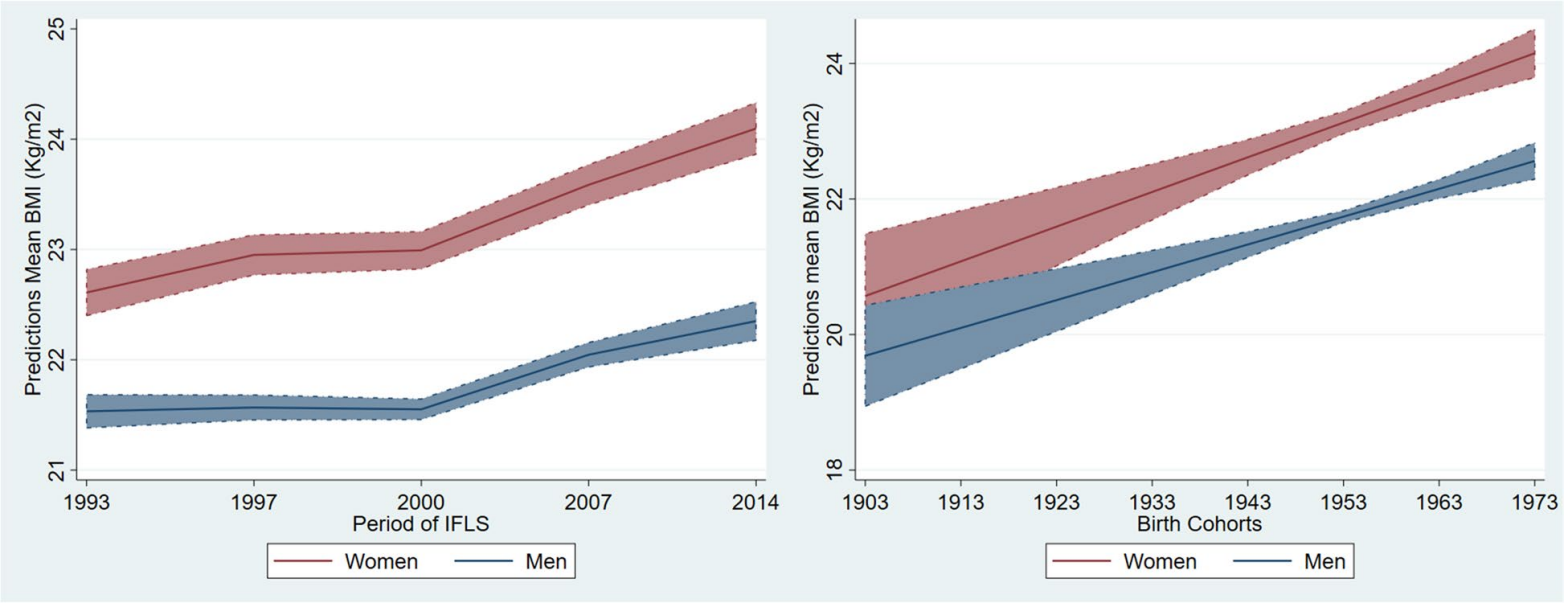
**a** and (**b**) Predicted period and cohorts effect to population mean BMI with 95% CI

Similar with the first analysis, the results in Table 4 find significant linear and quadratic effects of age on BMI changes for women and men, meaning individual BMI changes over age following a curvilinear function. Both models find different patterns in the effect of period from 1993 to 2014 to the changes of BMI in women and men. Compared to BMI in 1993, women’s BMI gradually rose over the period from 0.34 points in 1997 to 1.49 in 2014. For men, BMI was significantly higher only in 2007 and 2014, with increments of 0.51 and 0.82 points respectively. The significant effects of period suggest that changes in social and environmental context (or historical periods) from 1993 to 2014 contributed to the rise of population BMI in Indonesia. By centering birth cohort at the youngest cohort (born in 1973), both models show significant positive effects of birth cohort on individual BMI, meaning that more recently-born cohorts are heavier than previous cohorts. The effect of cohort prevails even after accounting for intra- and inter-birth cohort differences in the intercept of BMI as shown in the level three random effect. Predicted adjusted mean BMI when controlling the effect of period and cohort are illustrated in Fig. 2 (panel a and b).

For both women and men, the random effects show that considerable individual variability in intercept and slope remains after the inclusion of the growth model (fixed effects). The large magnitude of variance around the intercept terms suggests that individual differences in initial BMI play a major role in determining BMI growth over time. The random model indicates that most of the variance in mean BMI (intercept) comes from the individual level (10.99) rather than birth cohort level (0.16). These results suggest fairly significant variation between birth cohorts in mean BMI.

### Educational gradient of BMI trajectories across cohorts for women and men

Table 3 illustrates the effect of education on women’s and men’s BMI. Education attainment has substantially different impacts on the BMI of women and men. In the women’s model, the main effect of education on BMI diminishes after interacting education with cohort, as well as when age is included in the model. This suggests that the effect of education on women’s BMI depends on age (life-course) and individual birth cohort. Among women, inequality in mean BMI across the educational spectrum is wider over life-course. The negative interaction between education and cohort reveals that women from younger birth cohorts with high level of education tend to have lower BMI compared to low educated women. It also shows that women with higher educational attainment have lower BMI compared to previous generations of women with the same education level. Predicted margin from the model shows that having tertiary education lowers the risk of weight gain (-0.04 point) among recently-born cohorts of women.The significant negative interaction between education and cohort to BMI suggests that the lower rate of weight gain among younger cohorts is associated with the increased level of educational attainment achieved by individuals in the same birth cohorts.

The model estimating the effect of education for men differs slightly from the women’s model, as it lacks an interaction between education and age due to convergence issues. This likely stems from the smaller sample of male panel respondents as compared to women. Further, the men’s model in Table 4 shows almost zero variation at cohort level. This means that most variance at cohort level has been explained by covariates in the model, so it is not necessary to include interaction between age and education in the men’s model. Thus, the men’s model only tests the main effect of education and whether the effect of education on BMI varies across birth cohorts. Having secondary or tertiary education is associated with a higher BMI among men, with increments of 0.83 for men with secondary education and 1.91 for tertiary education. Among the recently-born cohort of men, having tertiary education still increases the risk of weight gain by 0.04 point. Over time, the negative effect of higher education on the change in BMI is also observed in the men of the younger cohorts, although at a much smaller magnitude compared to women.

**Fig. 3:**
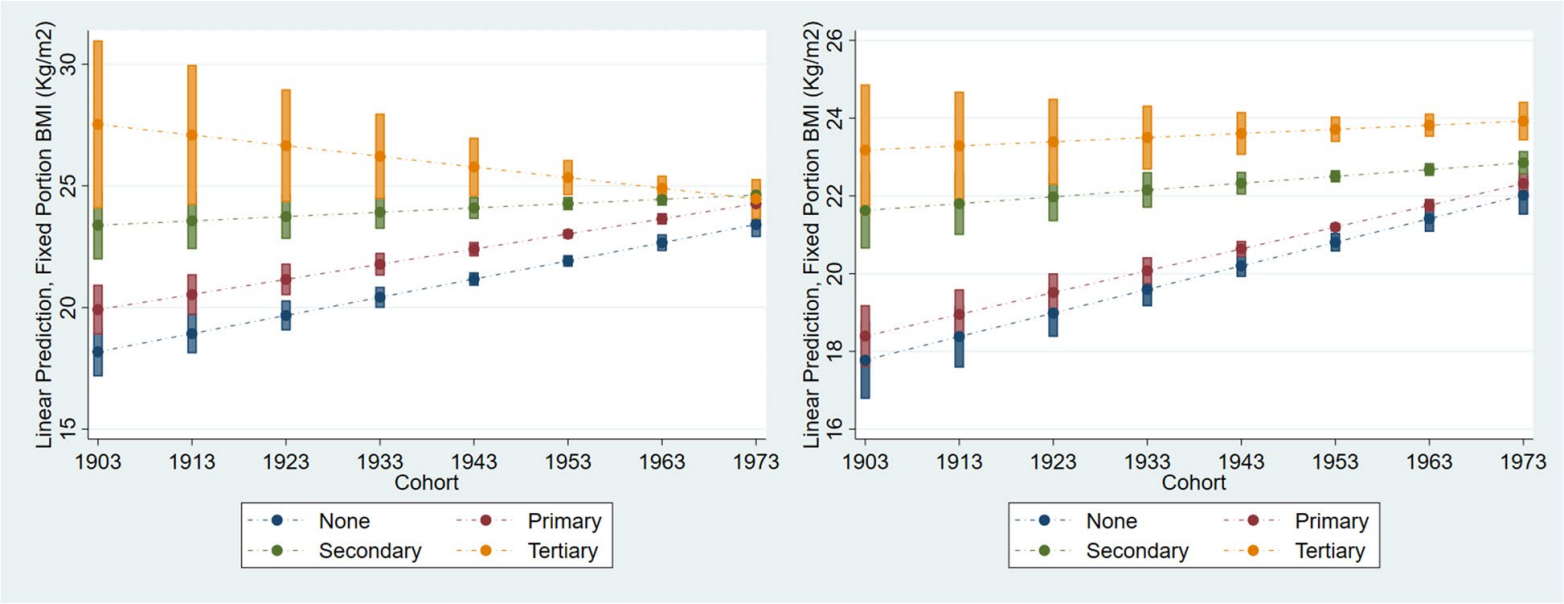
The effect of education attainment to mean BMI for women (**a**) and men (**b**) with 95% CI

Predicted adjusted mean BMI by educational spectrum across cohorts is illustrated in Fig. 3. This figure shows the shifting effect of educational attainment on BMI across cohorts of men and women in Indonesia. Panel a of Fig. 3 shows that inequalities in mean BMI across educational spectrum were wide among the older generation of women, but diminish among younger generations. Among older generations, women with lower education have mean BMI around underweight to normal BMI cut-offs (< 23 kg/m^2^ for Asia–Pacific BMI cut-off). However, among the younger generation, the mean BMI of women with low education attainment are now at in the overweight classification of BMI (23–24.9 kg/m^2^ for Asia–Pacific BMI). This suggests that the risk of overweight is more dispersed across the educational spectrum among more recently-born cohorts of women. The mean BMI of women with tertiary education shows a downward trend over cohorts, from obese (≥ 25 kg/m^2^) among older cohorts to overweight in younger cohorts. These trends suggest that, among younger cohorts, tertiary education is acting as a “social vaccine” to growth of BMI and obesity for women.

The educational gradient of mean BMI among men (Panel b of Fig. 3) also narrows over cohort, but the gradient persists in younger birth cohorts. The predicted margin of mean BMI for men across the educational spectrum show that, in contrast to its protective effects among women, tertiary education is associated with weight gain among men in younger cohorts and tend to be overweight. Although the mean BMI of men with secondary education or lower increases over time, the mean BMI of these groups remain in the normal BMI range (≤ 23 kg/m^2^).

## Discussion

Using panel data from the Indonesian Family Life Survey, this study provides evidence on how social inequalities in BMI are shifting over time and across cohorts in a low-middle income setting. Analyses of both men’s and women’s BMI trajectories finds that inequality in BMI by educational attainment has narrowed among younger generations, to the point of diminishing entirely among recent cohorts of women. Interestingly, this study finds a differential direction of the association between tertiary education and mean BMI between men and women. Having tertiary educational attainment after the age of 20 is a protective factor for overweight and obesity for women in younger cohorts. However, men with tertiary education remain heavier compared to low-educated people over generations. Sex differences in higher education’s effect on obesity are also observed in Korea despite it have been found to be a protective factor for both sexes in most developed countries [18].

Based on the literature, there are several possible explanations for the educational gradients identified in this study. Firstly, the narrowing gap in BMI by education attainment in Indonesia may be the result of increasing access to and quality of education over time. Inequality in access to education in Indonesia has decreased over time, as younger generations enjoy better access to education [41]. Secondly, declining inequality in mean BMI across the educational spectrum may be driven by the ongoing nutritional transition in the country, characterized by rising access to western diets and processed foods containing high fat, salt and sugar affecting the entire population [49]. According to the population education transition hypothesis, a positive association between higher education and overweight/obesity is likely to be observed at the onset of the nutritional transition [20] because high-educated people have better access to food. As the population becomes more widely exposed during the nutritional transition and the health risks of obesity become profound, a negative association between education and obesity emerges [20] because high-educated people are more aware of the risks of obesity for health, and more capable of maintaining optimal weight compared to socially disadvantaged people.

Thirdly, the different effect of tertiary education on BMI for men and women suggests that the present sex differences in weight gain are passing through education. For women, the negative effect of tertiary education on weight gain may reflect higher access to information and knowledge that help individuals maintain their body weight [50]. Higher education is also related to better socioeconomic status, which provides advantages for people to access healthy food and pursue healthy lifestyles. The protective effect of education on health risk behaviors among women supports the “ceteris paribus” or “social vaccine” hypothesis/premise [20]. Social vaccine is an approach to prevent the effect of economic and social structural, political and environmental contexts causing people or communities to become vulnerable to diseases that leads to disparities in population health [51]. The approach places emphasis on tackling the social determinants of health, for example addressing disparities in access to education to prevent health risk behaviours or diseases. The present study provides evidence on the important role of greater educational attainment to prevent the risk of weight gain, especially among women in Indonesia. If current trends in the educational gradient in BMI among Indonesian women continue, we may observe an inverse association between educational attainment and BMI in the near future, where women with high education are at lower risk of overweight/obesity. Further, different food consumption patterns and physical activity by sex may explain the different effect of high levels of education to the risk of obesity for men and women in Indonesia [38].

Another important contribution of this study is the examination of age-period-cohort (APC) effects on BMI change in Indonesia. The results suggest that both biological and historical contexts simultaneously explain the change in BMI among Indonesians. This study reveals that the main effect of age is to determine the individual rate of increase in BMI, and the rate of age-related declines in BMI during the aging process. The APC analysis reveals significant cohort effects, with younger generations tending to have higher BMI compared to older generations. This suggests that historical conditions embedded in certain birth cohorts shape differences in BMI trajectory over adulthood and across cohorts.

The APC analysis also reveals that the period of surveys from 1993–2014 (5 waves) also have significantly different effects on population mean BMI. The mean BMI of Indonesia increased slowly from 1993 to 1997, then flattened between 1997 and 2000, before increasing sharply in 2007 and 2014. These period fluctuations may be related to the broader macro-economic changes occurring in Indonesia during these years. Indonesia experienced high rates of Gross Domestic Product (GDP) growth from 1990–96 [52]. In the following years, the country’s economy plunged due to the East Asian economic crisis of 1997–98, causing increase unemployment, poverty, and declining income in the country [52]. Post crisis, the country experienced stable growth of GDP and declines in the rate of poverty over time [52]. Kinge et al. [53] argue that GDP has positive association with obesity levels and the association might be mitigated by education.

### Public health significance

This study provides important information and knowledge that can inform health policy related to increases in overweight/obesity in Indonesia. The findings show that women are at high risk of experiencing overweight/obesity after age 40. Hence, prevention programs can focus on assisting women in early adulthood to maintain normal weight. Obesity prevention programs for women also can be integrated into child and maternal health programs in Indonesia. For example, a potential strategy could be to monitor women’s BMI during and after pregnancy to prevent obesity among women in reproductive age. As younger cohorts tend to have higher BMI at early adulthood compared to previous generations, prevention programs also need to target younger generations at earlier ages. When developing policies and intervention programs, the government may focus on intervention strategies to limit the impact of obesogenic environments during the nutritional transition. Prevention programs should consider obesity inequalities across population groups to ensure that prevention strategies are able to reduce inequalities. Obesity prevention programs also need to focus on low educated men and women to prevent rising trends of obesity among these socially disadvantaged groups in the future. Increasing access to higher education and nutrition education may mitigate the obesogenic changes to the risk of obesity during nutritional transition in the country.

### Strengths and limitation of this study

Analysis based longitudinal data is required to disentangle the effects of age, period, and cohort to reduce bias and improve robust estimation. The findings reveal important evidence on the actual effects of APC to BMI change in the country that can help inform better health policy to prevent future increases of overweight/obesity in Indonesia. This study confirm that the expected shifting in educational gradients in obesity postulated by Baker et al. [20] are occurring in Indonesia, and suggests that this may also happen in other low middle income countries. The analytical approach in this study can be replicated in other low-middle income country settings to extend knowledge on obesity patterns in countries that remain understudied.

Despite the strengths, this study only focuses on examining social inequality in BMI trajectory based on a time-invariant measure of educational attainment. The substantial residual variation remaining around the individual intercept suggests that the inclusion of additional covariates could improve explanation of inter-individual differences in BMI trajectories. Further analysis including time-variant variables such as changes in wealth index, income, or occupations can extend knowledge on the effect of the changes in economic status on BMI trajectories over the life course. There might be selection bias in this study as result of attrition due to mortality among the oldest birth cohorts or drop-out among the youngest cohorts with high mobility, and sample restrictions that exclude individuals with extremes of height and weight or who have less than three BMI measures during the ILFS period. However, this study analyses IFLS panel data that has a 86.9% interview rate in all 5 waves suggesting lower risk of bias from non-random attrition. Further, sensitivity analysis on the models (supplemental Table 3) shows estimation including all observations with a minimum of one BMI measure shows consistent results.

The random effects in the estimation model find a wide variance of BMI intercept in this study. This suggests that further in-depth analysis is needed to investigate the different effects of initial BMI to the risk of obesity. The investigation of BMI trajectories in this study provides insights for future analysis exploring the effect of BMI trajectories for later-life health outcomes in Indonesia.

## Conclusion

In conclusion, this study identified different BMI trajectories between men and women in Indonesia, with the rate of weight gain higher among women compared to men. Age, period and cohort factors independently contribute to the increase of BMI in Indonesia. The gap in mean BMI by educational levels is narrowing over cohorts in the Indonesian population, as the mean BMI of people with low education increases gradually across younger birth cohorts. Tertiary education has a protective effect on obesity among women in younger generations. Though having tertiary education is still associated with weight gain among men, this rate of BMI increase is much lower than for men with less schooling. These rising trends in overweight/obesity among low-educated people are a substantial concern for the future of public health and non-communicable disease risk in Indonesia, as these socially disadvantaged groups already face substantial barriers to receiving health care and effective treatment. Finally, the potential interaction between improvements in Indonesian life expectancy and the rise in mean BMI on successive cohorts over time may continue to increase the burden of overweight/obesity in the country.

## Supplementary Information

**Additional file 1: Supplementary Table 1.** Selection of observations. **Supplementary Table 2.** Participation rates for each birth cohort based on number of available BMI measures. **Supplementary Table 3.** Sensitivity analysis inclusion individual with minimum 1 BMI measures (model 1) and 3 BMI measures (model 2). **Supplementary Table 4.** Distribution of Overweight/ Obesity Based on Respondents’ Characteristics Measured on Each Wave of IFLS (*N*=14,810). **Supplementary Table 5.** Model Specification using HAPC for Women. **Supplementary Table 6.** Model Specification using HAPC for Men.

## Data Availability

The data used in these analysis are publically available from the RAND corporation IFLS repository at https://www.rand.org/labor/FLS/IFLS.html
