# Supplementary material for: Shifting educational gradients in body mass index trajectories of Indonesians: an age period cohort analysis
Source: BMC Public Health. 2022 May 18;22:1004. doi: 10.1186/s12889-022-13379-3 (PMC9115941; doi:10.1186/s12889-022-13379-3)
Supplement: Supplementary file 1 — Additional file 1: Supplementary Table 1. Selection of observations. Supplementary Table 2. Participation rates for each birth cohort based on number of available BMI measures. Supplementary Table 3. Sensitivity analysis inclusion individual with minimum 1 BMI measures (model 1) and 3 BMI measures (model 2). Supplementary Table 4. Distribution of Overweight/ Obesity Based on Respondents’ Characteristics Measured on Each Wave of IFLS (N=14,810). Supplementary Table 5. Model Specification using HAPC for Women. Supplementary Table 6. Model Specification using HAPC for Men. [file 12889_2022_13379_MOESM1_ESM.docx]

# Supplementary Materials (Appendix)

Supplementary Table 1: Selection of observations

|  | Wave | | | | | |
| --- | --- | --- | --- | --- | --- | --- |
|  | I | II | III | | IV | V |
| Total individual age >=20 on each wave before exclude outlier | 16,715 | 20,953 | 25,401 | | 34,119 | 40,835 |
| Obs. with extreme weight or height | 29 | 9 | 14 | | 205 | 17 |
| % outlier | 0.17% | 0.04% | 0.06% | | 0.60% | 0.04% |
| Total observations after excluding outlier with minimum 1 BMI measures | 16,686 | 20,944 | 25,387 | | 33,914 | 40,818 |
| Total observations included in analysis (born before 1974 and with minimum 3 BMI measures). Total =14,810 panel respondents | 10,452 | 12,595 | 14,082 | 12,779 | | 10,623 |

Supplementary Table 2: Participation rates for each birth cohort based on number of available BMI measures

| ***Cohort - 10 years interval (p-value <0.001)*** | **% observations based on Number of BMI measures** | | | | |  |
| --- | --- | --- | --- | --- | --- | --- |
|  | **1** | **2** | **3** | **4** | **5** | **At least 3 BMI measures** |
| 1964-1973 | 20.19 | 19.65 | 21.6 | 22.77 | 15.79 | 60.16 |
| 1954-1963 | 12.59 | 10.83 | 14.73 | 22.99 | 38.86 | 76.58 |
| 1944-1953 | 11.17 | 10.4 | 15.75 | 23.1 | 39.59 | 78.44 |
| 1934-1943 | 12.96 | 13 | 21.27 | 25.3 | 27.46 | 74.03 |
| <=1933 | 29.5 | 18.87 | 26.21 | 17.17 | 8.24 | 51.62 |

Supplementary Table 3: Sensitivity analysis inclusion individual with minimum 1 BMI measures (model 1) and 3 BMI measures (model 2)

|  | (Model 1) min 1 BMI measure | (Model 2) min 3 BMI measures |
| --- | --- | --- |
| # of obs | 102,933 | 60,531 |
| # of groups | 41,167 | 14,810 |
| **Fixed effect** |  |  |
| Mean BMI (intercept) | 20.00 | 19.22 |
| Rate of change by age - Centering age at 20 | 0.17 | 0.16 |
| Changing in rate by age | -0.002 | -0.002 |
| Sex-Female | 0.48 | 0.12 |
| Interaction Female-Age | 0.07 | 0.07 |
| Interaction Female - Age square | -0.001 | -0.001 |
|  |  |  |
| **Random effect** |  |  |
| Level I - within person |  |  |
| variance residual | 3.13 | 2.75 |
| Levell II - between person |  |  |
| variance initial BMI (intercept) | 10.29 | 9.28 |
| variance rate of change (slope) | 0.01 | 0.01 |
| Cov (intercept & slope) | -0.02 | -0.07 |
| p-value random effect |  |  |
|  |  |  |
| **Goodness of fit** |  |  |
| Log likelihood | -257715.12 | -143188.79 |
| AIC | 515450.2 | 286397.6 |
| BIC | 515545.7 | 286487.7 |

Supplementary Table 4. Distribution of Overweight/ Obesity Based on Respondents’ Characteristics Measured on Each Wave of IFLS (N=14,810)

|  | Wave I | | Wave II | | Wave III | | Wave IV | | Wave V | |
| --- | --- | --- | --- | --- | --- | --- | --- | --- | --- | --- |
|  | Overweight | Obese | Overweight | Obese | Overweight | Obese | Overweight | Obese | Overweight | Obese |
| *Sex* |  |  |  |  |  |  |  |  |  |  |
| Female | 23.84 | 7.96 | 26.8 | 10.84 | 29.13 | 12.63 | 32.38 | 19.49 | 35.45 | 25.66 |
| Male | 17.26 | 3.28 | 19.42 | 4.07 | 21.07 | 5.16 | 26.57 | 9.32 | 31.01 | 11.53 |
| *Age Group* |  |  |  |  |  |  |  |  |  |  |
| 20-29 | 18.6 | 2.79 | 19.75 | 4.56 | 21.52 | 5.97 | 0 | 14.29 |  |  |
| 30-39 | 23.38 | 7.01 | 27.26 | 7.96 | 27.72 | 9.15 | 32.43 | 16.18 | 35.45 | 23.64 |
| 40-49 | 25.06 | 8.25 | 27.72 | 11.89 | 30.1 | 12.49 | 33.74 | 18.24 | 37.7 | 24.18 |
| 50-59 | 19.61 | 5.92 | 21.87 | 7.84 | 26.18 | 9.87 | 31.11 | 16.36 | 34.96 | 22.13 |
| ≥60 | 13.79 | 3.99 | 15.56 | 5.28 | 16.26 | 5.59 | 20.76 | 7.75 | 26.76 | 10.39 |
| *Cohort* |  |  |  |  |  |  |  |  |  |  |
| 1964 -1973 | 18.6 | 2.79 | 22.75 | 5.53 | 25.18 | 7.66 | 33.14 | 17.3 | 38.03 | 24.18 |
| 1954 -1963 | 23.38 | 7.01 | 28.01 | 10.19 | 30.27 | 12.43 | 33.19 | 17.71 | 34.11 | 21.92 |
| 1944-1953 | 25.06 | 8.25 | 25.95 | 10.38 | 28.82 | 10.79 | 28.54 | 13.84 | 30.92 | 13.16 |
| 1934-1943 | 19.61 | 5.92 | 20.32 | 6.97 | 20.2 | 7.59 | 21.09 | 8.11 | 22.2 | 6.11 |
| ≤ 1933 | 13.79 | 3.99 | 14.43 | 4.81 | 13.87 | 4.2 | 15.57 | 4.5 | 15.21 | 2.91 |
| *Highest education attainment* |  |  |  |  |  |  |  |  |  |  |
| Never/Not completed primary education | 15.52 | 3.62 | 16.09 | 4.76 | 17.33 | 5.07 | 20.85 | 7.93 | 22.68 | 10.34 |
| Primary | 19.23 | 5.34 | 22.49 | 7.77 | 24.23 | 8.76 | 28.08 | 14.04 | 31.55 | 18.2 |
| Secondary | 28.43 | 8.13 | 29.41 | 9.63 | 30.71 | 11.5 | 35.09 | 18.75 | 39.2 | 23.5 |
| Tertiary | 30.79 | 13.9 | 33.94 | 12.45 | 36.93 | 15.51 | 44.3 | 23.27 | 46.34 | 28.75 |
| Notes: Distribution based on individuals who were measured on each waves | | | |  |  |  |  |  |  |  |

Supplementary Table 5. Model Specification using HAPC for Women

|  | **Baseline model** | **Model 1** | **Model 2** | **Model 3** | **Model 4** | **Model 5** |
| --- | --- | --- | --- | --- | --- | --- |
| # of obs | **33,334** | | | | | |
| # of groups | **8,003** | | | | | |
| # cohort group | **70** | | | | | |
| **Fixed effect** |  |  |  |  |  |  |
| *Mean BMI (intercept)* | 22.3 | 20.35 | 20.63 | 19.20 | 19.74 | 19.95 |
| *Rate of change by age - Centering age at 20* |  | 0.23 | 0.24 | 0.24 | 0.23 | 0.21 |
| *Changing in rate by age (age^2) - aging effect* |  | -0.003 | -0.003 | -0.003 | -0.003 | -0.003 |
| *Cohort - centering at youngest cohort* |  | 0.06 | 0.08 | 0.06 | 0.08 | 0.07 |
| *Period (wave of survey)* |  |  |  |  |  |  |
| 1993 |  |  |  |  |  |  |
| 1997 |  | 0.32 | 0.32 | 0.32 | 0.33 | 0.34 |
| 2000 |  | 0.36 | 0.36 | 0.36 | 0.38 | 0.38 |
| 2007 |  | 0.98 | 0.95 | 0.96 | 0.99 | 0.98 |
| 2014 |  | 1.52 | 1.48 | 1.49 | 1.54 | 1.49 |
| *Education* |  |  |  |  |  |  |
| None |  |  |  |  |  |  |
| Primary |  |  |  | 1.19 | 0.75 | 0.70 |
| Secondary |  |  |  | 1.69 | 0.57 | 0.21 |
| Tertiary |  |  |  | 1.80 | 0.01 | -0.61 |
| *Education#cohort* |  |  |  |  |  |  |
| None |  |  |  |  |  |  |
| Primary |  |  |  |  | -0.02 | -0.01 |
| Secondary |  |  |  |  | -0.08 | -0.06 |
| Tertiary |  |  |  |  | -0.15 | -0.12 |
| *Education#Age* |  |  |  |  |  |  |
| None |  |  |  |  |  |  |
| Primary |  |  |  |  |  | 0.01 |
| Secondary |  |  |  |  |  | 0.04 |
| Tertiary |  |  |  |  |  | 0.06 |
| **Random effect** |  |  |  |  |  |  |
| Level I - within person |  |  |  |  |  |  |
| variance residual | 5.6 | 4.30 | 3.28 | 3.27 | 3.27 | 3.27 |
| Levell II - between person |  |  |  |  |  |  |
| variance initial BMI (intercept) | 13.0 | 13.25 | 11.12 | 11.27 | 11.07 | 10.99 |
| variance rate of change (slope) |  |  | 0.01 | 0.01 | 0.01 | 0.01 |
| cov(intercept & slope) |  |  | -0.12 | -0.13 | -0.13 | -0.12 |
| Level III-between cohorts | 1.9 | 0.42 | 0.33 | 0.22 | 0.17 | 0.16 |
|  |  |  |  |  |  |  |
| **Goodness of fit** |  |  |  |  |  |  |
| Log likelihood | -85545.88 | -82085.10 | -80993.21 | -80913.42 | -80882.65 | -80852 |
| AIC | 171099.8 | 164192.2 | 162012.4 | 161858.8 | 161803.3 | 161748 |
| BIC | 171133.4 | 164284.7 | 162121.8 | 161993.5 | 161963.2 | 161933.1 |
| Likelihood-ratio test for model comparison |  | <0.001 | <0.001 | <0.001 | <0.001 | <0.001 |

Supplementary Table 6. Model Specification using HAPC for Men

|  | **Baseline model** | **Model 1** | **Model 2** | **Model 3** | **Model 4** | **Model 5** |
| --- | --- | --- | --- | --- | --- | --- |
| # of obs | **27,197** | | | | | |
| # of groups | **6,816** | | | | | |
| # cohort group | **72** | | | | | |
| **Fixed effect** |  |  |  |  |  |  |
| *Mean BMI (intercept)* | 21.09 | 19.99 | 20.19 | 20.22 | 19.01 | 19.40 |
| *Rate of change by age - Centering age at 20* |  | 0.21 | 0.16 | 0.17 | 0.17 | 0.17 |
| *Changing in rate by age (age^2) - aging effect* |  | -0.003 | -0.00241 | -0.00245 | -0.002 | -0.002 |
| *Cohort - centering at youngest cohort* |  | 0.10 | 0.05 | 0.06 | 0.05 | 0.06 |
| *Period (wave of survey)* |  |  |  |  |  |  |
| 1993 |  |  |  |  |  |  |
| 1997 |  |  | 0.06 | 0.02 | 0.03 | 0.03 |
| 2000 |  |  | 0.06 | -0.005 | 0.01 | 0.02 |
| 2007 |  |  | 0.60 | 0.46 | 0.49 | 0.51 |
| 2014 |  |  | 0.94 | 0.75 | 0.78 | 0.82 |
| *Education* |  |  |  |  |  |  |
| None |  |  |  |  |  |  |
| Primary |  |  |  |  | 0.48 | 0.30 |
| Secondary |  |  |  |  | 1.54 | 0.83 |
| Tertiary |  |  |  |  | 2.73 | 1.91 |
| *Education#cohort* |  |  |  |  |  |  |
| None |  |  |  |  |  |  |
| Primary |  |  |  |  |  | -0.005 |
| Secondary |  |  |  |  |  | -0.04 |
| Tertiary |  |  |  |  |  | -0.05 |
| **Random effect** |  |  |  |  |  |  |
| Level I - within person |  |  |  |  |  |  |
| variance residual | 3.23 | 2.71 | 2.69 | 2.07 | 2.07 | 2.07 |
| Levell II - between person |  |  |  |  |  |  |
| variance initial BMI (intercept) | 8.42 | 8.60 | 8.57 | 6.56 | 6.74 | 6.59 |
| variance rate of change (slope) |  |  |  | 0.01 | 0.01 | 0.01 |
| Cov (intercept & slope) |  |  |  | -0.06 | -0.08 | -0.07 |
| Level III-between cohorts | 1.24 | 0.04 | 0.04 | 0.02 | 0.01 | 4.09E-12 |
|  |  |  |  |  |  |  |
| **Goodness of fit** |  |  |  |  |  |  |
| Log likelihood | -62846.99 | -61006.93 | -60927.60 | -60037.25 | -59851.41 | -59833.76 |
| AIC | 125702 | 122027.9 | 121877.2 | 120100.5 | 119734.8 | 119705.5 |
| BIC | 125734 | 122085.3 | 121967.5 | 120207.2 | 119866.2 | 119861.5 |
| Likelihood-ratio test for model comparison |  |  | <0.001 | <0.001 | <0.001 | <0.001 |

Notes: All available cohorts are used in this analysis, so there are differences in number of cohort groups between women (70 groups) and men (72 groups). Using 70 cohort groups for men model, the results still consistent with model for 72 birth cohorts. So we decide to include all birth cohort for men.
